# Revealing Fungal Communities in Alpine Wetlands through Species Diversity, Functional Diversity and Ecological Network Diversity

**DOI:** 10.3390/microorganisms8050632

**Published:** 2020-04-27

**Authors:** Fei Xie, Anzhou Ma, Hanchang Zhou, Yu Liang, Jun Yin, Ke Ma, Xuliang Zhuang, Guoqiang Zhuang

**Affiliations:** 1 Research Center for Eco-Environmental Sciences, Chinese Academy of Sciences, Beijing 100085, China; feixie_st@rcees.ac.cn (F.X.); hczhou_st@rcees.ac.cn (H.Z.); liangyu181@mails.edu.ac.cn (Y.L.); junyin_st@rcees.ac.cn (J.Y.); make18@mails.ucas.ac.cn (K.M.); xlzhuang@rcees.ac.cn (X.Z.); 2 College of Resources and Environment, University of Chinese Academy of Sciences, Beijing 100049, China; 3 Sino-Danish College, University of Chinese Academy of Sciences, Beijing 101400, China

**Keywords:** fungi, ITS, functional diversity, ecological network, alpine wetland, Qinghai-Tibet Plateau

## Abstract

The biodiversity of fungi, which are extremely important in maintaining the ecosystem balance in alpine lakeside wetlands, has not been fully studied. In this study, we investigated the fungal communities of three lakeside wetlands from different altitudes in the Qinghai–Tibet Plateau and its edge. The results showed that the fungi of the alpine lakeside wetland had higher species diversity. Functional annotation of fungi by FUNGild software showed that saprophytic fungi were the most abundant type in all three wetlands. Further analysis of the microbial phylogenetic molecular ecological network (pMEN) showed that saprophytic fungi are important species in the three wetland fungal networks, while symbiotic fungi and pathotrophic fungi have different roles in the fungal networks in different wetlands. Community diversity was high in all three lakeside wetlands, but there were significant differences in the composition, function and network structure of the fungal communities. Contemporary environmental conditions (soil properties) and historical contingencies (geographic sampling location) jointly determine fungi community diversity in this study. These results expand our knowledge of fungal biodiversity in the alpine lakeside wetlands.

## 1. Introduction

The alpine wetland is one of the important wetland types, and it is a key area for protecting precious species resources, maintaining ecological balance and biodiversity in the plateau region. Fungi are an essential group of microbial species in the ecosystem [1]. They are important components of material cycling and energy flow in the ecosystem. They are widely distributed in various terrestrial environments such as forests [2,3], grasslands [4,5], hot springs [6,7], permafrost [8,9] and marsh wetlands [10,11]. Fungi have different species composition and distribution patterns in diverse habitats, thus forming complex fungal communities [12,13,14,15,16].

A group of species that utilize the same class of environmental resources in a similar way, whether related or unrelated, is considered a functional group (also referred to as “guild”) [17,18]. Soil fungal communities are composed of multiple fungi. Based on their trophic strategy, soil fungi are divided into three functional groups: saprotrophs, symbiotrophs and pathogens [17]. Saprophytic fungi are the primary decomposers in the soil and play an essential role in regulating the flow of carbon and nitrogen [12,13,14,16,19]. Symbiotrophs are also important in ecosystems, especially mycorrhizal fungi. It is estimated that 92% of the plants on the earth can form a symbiotic relationship with mycorrhizal fungi, the symbiotic cooperation between mycorrhizal fungi and plants promotes the growth of plants and enhances plant resistance [20,21,22]; and at the same time, fixes the nutrient source obtained from plants to the soil, which improves soil nutritional conditions [23]. Pathogenic fungi assist in the decomposition of animals, plants and microorganisms and can even cause death [24,25,26,27,28]. The combination of these different trophic types of fungi will form a functional diversification of fungal communities in soil, which will affect the soil ecosystem process and is important for maintaining the health of the ecosystem [20].

The cooperation or competition for environmental resources and space means the microorganisms in the soil never live in isolation but form a complicated network of ecological relationships [29,30,31]. Phylogenetic molecular ecological network (pMEN) analysis provides a good method for studying the interactions between different populations in a community [32,33]. The network topology, module memberships and the phylogenetic relationships of the node (population) will distinguish whether they are key microbes in the ecological network [31]. The key species are important groups that have a significant impact on the community or even drive the covariation of the community [31]. Therefore, identifying the key species of the fungal community and using functional annotation analysis can provide a better understanding of the structure and functional diversity of the fungal community.

Recent biogeographical studies of microorganisms have shown that contemporary environmental conditions and historical contingencies (geographic sampling location) are the main factors affecting fungal and bacterial communities in ecosystems [34,35]. The uplift of the Qinghai–Tibet Plateau changed the hydrothermal conditions in the region and formed a unique natural environment in the plateau. This unique environment has had a significant impact on local biology [36,37,38]. Specifically, a large number of lakes have been formed in the Qinghai–Tibet Plateau, the lake area of this region accounts for 52% of the country and a large number of alpine lakeside wetlands have formed [39], and the fungal communities in these wetlands have not been well-studied. To fill this gap, we studied alpine lakeshore wetlands at different altitudes to investigate the diversity of fungal communities in wetlands using Illumina high-throughput sequencing technology. FUNGuild software [17] and phylogenetic molecular ecological network (pMEN) analysis [31] were used to further analyze the functional diversity of fungal communities and the complex interactions between different fungi taxa. Finally, we analyzed the effects of geographic and environmental factors on the fungal community to determine the factors that drive the fungal community composition.

## 2. Materials and Methods

### 2.1. Study Area and Sample Collection

The research sites are the Huahu Lake wetland of Zoige national reserve wetland (N 33°56′14.7″, E 102°49′44.6″), Mangcuo Lake wetland (N 29°32′4.68″, E 98°50′7.48″) and Baima Snow Mountain nature reserve wetland (N 27°37′38.09″, E 99°07′59.80”). They are typical lakeside alpine wetlands located in the northeast, southeast of the Qinghai–Tibet Plateau and the middle of the Hengduan Mountains in the south of the Qinghai–Tibet Plateau, respectively. The altitudes of the three wetland sampling points were 3433 m, 4297 m and 3869 m. In August 2019, eight soil samples with soil depths of 0–20 cm below the soil surface were collected from each wetland. The soil samples were transported to the laboratory on ice in sterile bags, where they were stored at −20 °C.

### 2.2. Soil Properties Analyses

Soil pH was measured in situ with a portable multi-parameter water analyzer (WTW, Multi340i, Weilheim, Germany). The differences in mass of fresh soil dried at 105 °C for 24 h were calculated as the moisture content (MC). An AA3 continuous flow analytical system (Seal, Norderstedt, Germany) was used to determine ammonia nitrogen (AN) and nitrate nitrogen (NN). The total phosphorus (TP) was measured by the perchloric acid-concentrated sulfuric acid digestion and the Mo-Sb colorimetric method. The elemental analyzer (vario MACRO cube, Elementar, Hesse, Germany) was used to measure total carbon (TC) and total nitrogen (TN).

### 2.3. DNA Extraction, PCR Amplification and Sequence Data Preprocessing

DNA was isolated from soils using a Power Soil DNA isolation kit (QIAGEN, Hilden, Germany). The concentration of DNA was measured with a NanoDrop (NanoDrop 2000, Thermo Scientific, Waltham, United States). PCR amplifications were determined using universal primers ITS3F (5′-GCATCGATGAAGAACGCAGC-3′) and ITS4R (5′-TCCTCCGCTTATTGATATGC-3′) to amplify the ITS2 region of fungi. PCR reactions were performed in a 50 μL mixture containing 2× Premix Taq (25 μL), DNA template (3 μL), forward primer (1 μL), reverse primer (1 μL) and nuclease-free water (20 μL). The following procedure was used for amplification: 94 °C for 5 min, 25 cycles of 94 °C for 30 s, 55 °C for 30 s, followed by 72 °C for 30 s and a final extension at 72 °C for 16 min. The quality of the PCR products was measured by 1% agarose gel electrophoresis. Metagenomic sequencing libraries were constructed and checked for quality, then 250-bp paired-end sequencing was performed on Illumina MiSeq sequencing (Illumina, San Diego, CA, USA). Trimmomatic was used to trim the raw sequences reads with low base quality. FLASH was used to merge forward and reverse reads with at least 10-bp overlaps and less than 5% mismatches. The quality control process using QIIME (V1.8.0) was used to remove sequences that were shorter than 200 bp with an average quality score lower than 25 in the raw reads. The remaining sequences were clustered into operational taxonomic units (OTUs) at the 97% similarity level, and chimeras and singleton OTUs were removed using the UCHIME and Usearch. For each OTU, the most abundant read was designated as the representative sequence, and the taxonomic identity was annotated within the Unite Database using the QIIME software. All of the sequence data have been submitted to the GenBank Sequence Read Archives (http://www.ncbi.nlm.nih.gov) under BioProject ID PRJNA607162.

### 2.4. Statistical Analysis

Multiple diversities were used to analyze the alpha and beta diversity of the community. At the same time, the environmental factors driving community change were identified. IBM SPSS statistics 25 (IBM, Endicott, New York, NY, USA) was used to conduct general statistical analysis. FUNGuild software was used for an analysis of the function group (guild) of each OTU, and the relative abundance of the guild was obtained by dividing the number of sequences in a particular guild by the total sequence. The phylogenetic molecular ecological network (pMEN) graphs were constructed on the online analysis pipeline (http://ieg4.rccc.ou.edu/MENA/main.cgi) [31,32] and visualized using Cytoscape 3.6.1 software. Mantel and partial mantel test and multivariate regression tree analyses were used to analyze the effects of geographic sampling location and environmental factors on fungal communities.

## 3. Results

### 3.1. Geographical Parameters and Soil Properties

The altitude of the three lakeside wetlands was between 3433 and 4297 m, which is typical for alpine wetlands. As shown in Table 1, there were significant differences in soil physical and chemical properties among the three wetlands (Table 1). The soil in the Huahu Lake wetland was neutral, the soil in Mangcuo Lake wetland was more acidic, with an average pH of 5.69, and the Baima Snow Mountain wetland soil was weakly acidic. The moisture content (MC) of the Huahu Lake wetland was significantly higher than the other two wetlands. The Huahu Lake wetland was rich in peat reserves. Its total carbon and total nitrogen contents were 143.38 ± 24.26 g/kg dry soil and 11.27 ± 2.16 g/kg dry soil, respectively. The total carbon and total nitrogen in the Mangcuo Lake wetland, 46.97 ± 10.29 g/kg and 4.67 ± 1.01g/kg, respectively, were much lower than those in Huahu Lake wetland. The total carbon and total nitrogen of the Baima Snow Mountain wetland were the lowest, 24.25 ± 7.41 g/kg dry soil and 2.69 ± 0.65 g/kg dry soil, respectively. Soil total phosphorus content (TP) did not vary significantly between the three wetlands. Ammonia nitrogen (AN) was highest in the Baima Snow Mountain wetland (4.94 ± 1.13 mg/kg dry soil), and nitrate nitrogen (NN) was highest in the Mangcuo Lake wetland (1.75 ± 0.57 mg/kg dry soil). The C/N ratio was very similar among the three wetlands, with average values of 12.82, 10.25 and 8.90.

### 3.2. Fungal Community Composition

In total, 4,697,780 raw sequences were obtained for fungal communities in three wetlands. After the elimination of the sequences with poor quality, 4,415,617 high-quality fungal sequences were obtained from three wetlands. The 97% sequence-similarity cutoff yielded at least 15 phyla, 43 classes, more than 98 orders, 192 families and over 278 genera. These data indicate that fungal communities are abundant in alpine lakeshore wetlands at different altitudes. Figure S1 shows a comparison of the common and endemic species of the three wetlands using Venn diagrams. There were 1793 identical OTUs in the three wetlands. The Baima Snow Mountain wetland had the highest number of unique OTUs, 298. There were 286 unique OTUs for the Huahu Lake wetland and 131 unique OTUs for the Mangcuo Lake wetland.

**Figure 1:**
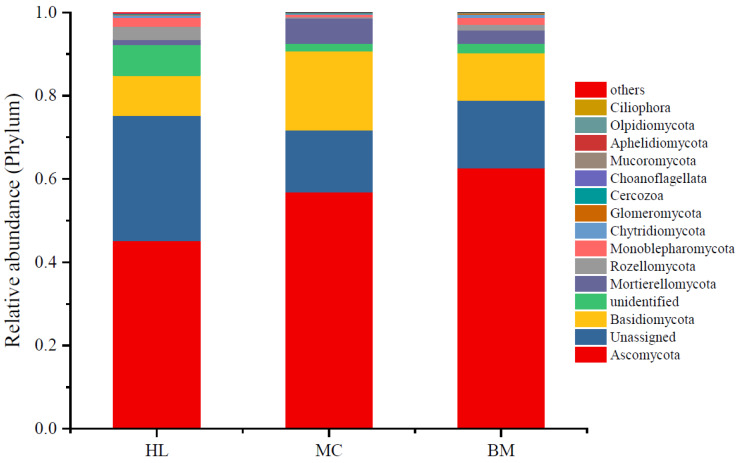
Fungal community variation in the three wetlands. The mean relative abundance of fungi from eight samples taken from each wetland. Each bar height represents the relative abundance, and color represents a particular fungal phylum. HL, MC and BM represent the Huahu Lake wetland, Mangcuo Lake wetland and Baima Snow Mountain wetland, respectively.

Statistics calculated the top 15 phyla of fungal relative abundance in each wetland (Figure 1). The fungi with the highest abundance were Ascomycota with relative abundances ranging from 0.45 to 0.63. Numerous sequences could not be classified into known fungi, especially the sequences for samples collected from the Huahu Lake wetland. Basidiomycota had a relative abundance of 0.10–0.16, which was the third-highest phylum by relative abundance. The relative abundance of phyla of Mortierellomycota, Rozellomycota and Monoblepharomycota was all higher than 0.01, while the relative abundances of the other nine minor phyla were lower than 0.01. The relative abundance of wetland fungi at the genus level is shown in Table S1. Among them, the relative abundance of fungi that have not been assigned to the genus is the highest, accounting for 0.78–0.86. The most abundant genus in Huahu wetland was *Phialophora*, followed by *Tricholoma*. There are six genera with relative abundances greater than 0.01 in Mangcuo Lake wetland, namely *Dimorphospora*, *Mortierella*, *Pilodema*, *Tricholoma*, *Chaetosphaeria* and *Coprinopsis*. The relative abundances in the Baima Snow Mountain Wetland are higher than 0.01, which are *Mortierella*, *Dimorphospora*, *Solicoccozyma*, *Neobulgaria* and *Tomentella*, respectively.

### 3.3. Fungal Community Diversity

**Figure 2:**
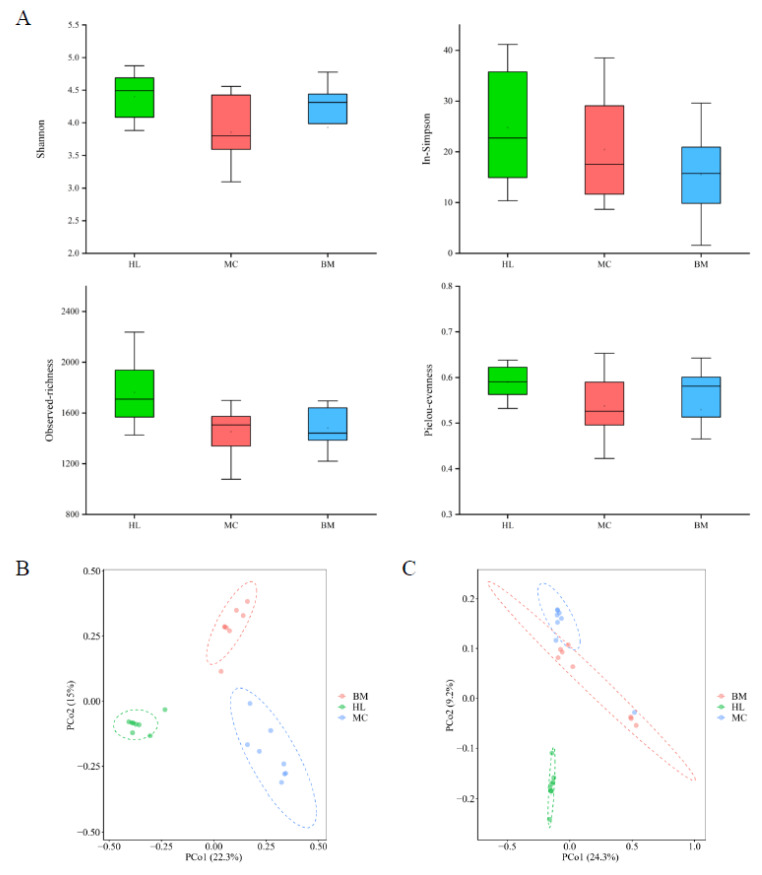
Alpha diversity (**A**) and principal coordinates analysis (PCoA) of fungal communities in the three wetland ecosystems. PCoA based on Bray–Curtis (**B**) and Jaccard (**C**) distance. HL, MC and BM represent the Huahu Lake wetland, Mangcuo Lake wetland and Baima Snow Mountain wetland, respectively.

A community diversity analysis was conducted to study the fungal community structure of the three wetlands. The results of alpha diversity analysis showed that the community richness, evenness and diversity of fungal communities were highest in the Huahu Lake wetland, next highest in the Baima Snow Mountain wetland and lowest in the Mangcuo Lake wetland (Figure 2A). The variation between the three wetlands in terms of alpha diversity at the OTU level was small. Except for the observed-richness index of the Huahu lake wetland, which was higher than the other two wetlands, there was no significant difference in the other indices.

The principal coordinates analysis (PCoA) based on Bray–Curtis (Figure 2B) and Jaccard (Figure 2C) distance showed significant structural differences among the fungal communities in the three wetland ecosystems. The difference between the Huahu Lake wetland and the other two wetlands is relatively large, while the distance between the Mangcuo Lake wetland and Baima Snow Mountain wetland is relatively small, indicating that the fungal community structure of Huahu Lake wetland is significantly different from the other two. Permutational multivariate analysis of variance (PERMANOVA) was used to quantify the structural differences of the fungal communities (Table S2). The fungal communities in the three wetlands were significantly different at the 0.001 level. A higher F-value represents a greater difference between the wetlands. The F-values between Huahu Lake wetland and the other two wetlands are 7.64 and 6.27, which are higher than the F-values between the other two wetlands. This result was consistent with the PCoA results.

Based on the above results, although the fungal communities in the three lakeside wetlands have little difference in alpha diversity, the community beta-diversity results show that there are significant differences in the fungal diversity of the three wetlands.

### 3.4. The Trophic Strategies of Fungal Communities

The above analysis is based on species richness or taxonomic identity. In order to further analyze the trophic strategies of the three wetland fungal communities [40], the annotation tool FUNGuild was used to analyze the trophic mode of the wetland fungi [17].

FUNGuild detected three major trophic modes and fifteen guilds among the three wetland fungal communities. Excluding the unassigned sequences and sequences that were identified to only kingdom level, guilds were successfully assigned to 39.63–43.07% of the OTUs in the fungal community dataset from the three wetlands. Simultaneously, guilds were assigned to 61.62–64.10% of the sequences in the fungal community dataset from the three wetlands (Table S3).

**Figure 3:**
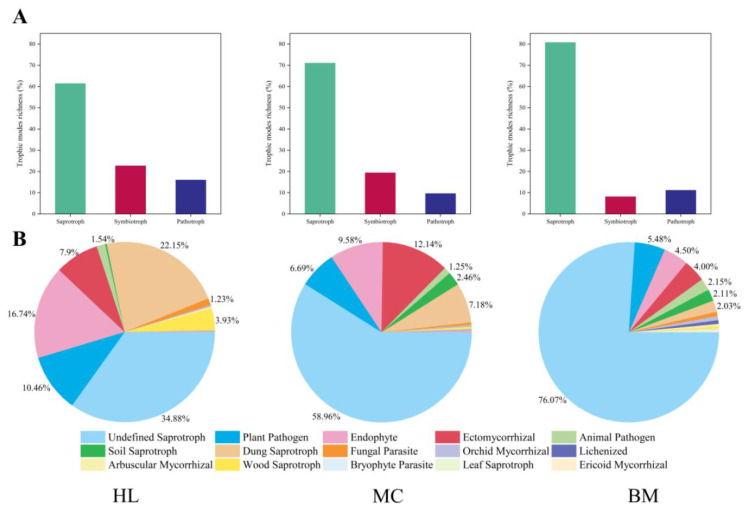
Guild assignments for three high-throughput wetlands datasets using FUNGuild software. (**A**) show the relative abundance of fungi of three trophic modes statistics based on sequence richness. (**B**) shows the relative abundance of fungi of 15 guilds statistics based on sequence richness. HL, MC and BM represent the Huahu Lake wetland, Mangcuo Lake wetland and Baima Snow Mountain wetland, respectively.

Relative abundance was calculated based on the number of sequences. The trophic mode proportions for the Huahu Lake wetland were 61.25% saprophytic, 13.49% pathological and 25.26% symbiotic. The Mangcuo Lake wetland was 69.06% saprophytic, 8.89% pathological and 22.05% symbiotic. The saprophytic type accounted for 80.78% of the fungal communities in the Baima Snow Mountain wetland, and the pathological and symbiotic types accounted for 8.86% and 10.36%, respectively (Figure 3A). There were relatively few saprophytic fungi in the Huahu Lake wetland fungal community but more in the Baima Snow Mountain wetland. The fungal communities in the Huahu Lake wetland and Mangcuo Lake wetland were higher in symbiotic and pathotrophic types than in other wetlands, but these types were fewer in the Baima Snow Mountain wetland. The results of the Pearson correlation analysis between the three trophic types showed that there was a very significant negative correlation between saprophytic and symbiotic fungi (*p* < 0.01; Table S4).

Undefined saprotrophs were the largest guild in sequence richness among the three wetland fungal communities (Figure 3B), but its proportion in the three wetlands varied: 34.88% in the Huahu Lake wetland, 58.96% in the Mangcuo Lake wetland and 76.07% in the Baima Snow Mountain wetland. Among the fungal communities in the Huahu Lake wetland, dung saprotroph was the second-largest guild. Endophyte (16.74%) and ectomycorrhizal (7.90%), which belong to the symbiotic trophic type, accounted for 24.64% of the entire sequence. The pathotrophic type was mainly plant pathogen, accounting for 10.46% of the whole sequence. The second and third largest guild fungal communities in the Mangcuo Lake wetland were symbiotic trophic ectomycorrhizal (12.14%) and endophyte (9.58%). Dung saprotroph and plant pathogen accounted for 7.18% and 6.69% of the entire sequence, respectively. The trophic modes of the fungal community in the Baima Snow Mountain wetland were significantly different from those of the other two wetlands. Among the top five of the guild sequence richness, undefined saprotroph (76.07%) accounted for a relatively high proportion. The pathotroph of plant pathogen (5.48%) and animal pathogen (2.15%) accounted for 7.63%. The symbiotic trophic endophyte and ectomycorrhizal accounted for 4.50% and 4.00% of the entire sequence, respectively.

### 3.5. Analysis of Fungal Ecological Network

According to the previous analysis, there are differences in the fungal community structure of the three wetlands. However, the relationship between fungi in different wetland communities is unclear. Therefore, we used the ecological network analysis at the genus level to study the interaction mode of fungi among wetland microbial communities. As previously described, the phylogenetic molecular ecological network (pMEN) of the three wetland fungal communities was based on random matrix theory (RMT) [31,32,41]. Illumina high-throughput sequencing data of eight samples from each wetland were used to construct molecular ecological networks (MENs). The fungal ecological networks of the three wetlands were named MEN_HL, MEN_MC and MEN_BM.

The topological characteristics of the fungal molecular networks from the three wetlands are shown in Table S5. The network sizes of the three wetlands were similar. The number of nodes for the MEN of the Huahu Lake wetland was 95; for the Mangcuo Lake wetland, it was 94 and for the Baima Snow Mountain wetland, it was 94. However, the number and type of links for the three wetlands varied: 81 negative and 86 positive links for the Huahu Lake wetland, 199 negative and 166 positive links for the Mangcuo Lake wetland and 131 negative and 75 positive links for the Baima Snow Mountain wetland. The average connectivity results showed that there were differences in the network complexity of the three wetlands. The average connectivity value of MEN_MC was a maximum of 7.77, indicating that the fungal network in the Mangcuo Lake wetland was more complicated than in other wetlands. The fungal network in Huahu Lake wetland was relatively simple, with an average connectivity value of 3.52. The average path distances of the three wetland fungal networks were 4.82 (Huahu Lake), 3.21 (Mangcuo Lake) and 4.23 (Baima Snow Mountain), indicating that the degree between genera in the network of Huahu Lake wetland was higher than the others and that of Mangcuo Lake wetland was the lowest. A module is a group of genera that are well linked among themselves but are less connected with genera belonging to other modules. The number of modules in fungal MEN of the Huahu Lake wetland, Mangcuo Lake wetland and Baima Snow Mountain wetland was 8, 5 and 10, respectively. The number of nodes in modules of MEN in Huahu Lake wetland and Baima Snow Mountain wetland was 2–21, while this value of Mangcuo Lake wetland was 3–32.

**Figure 4:**
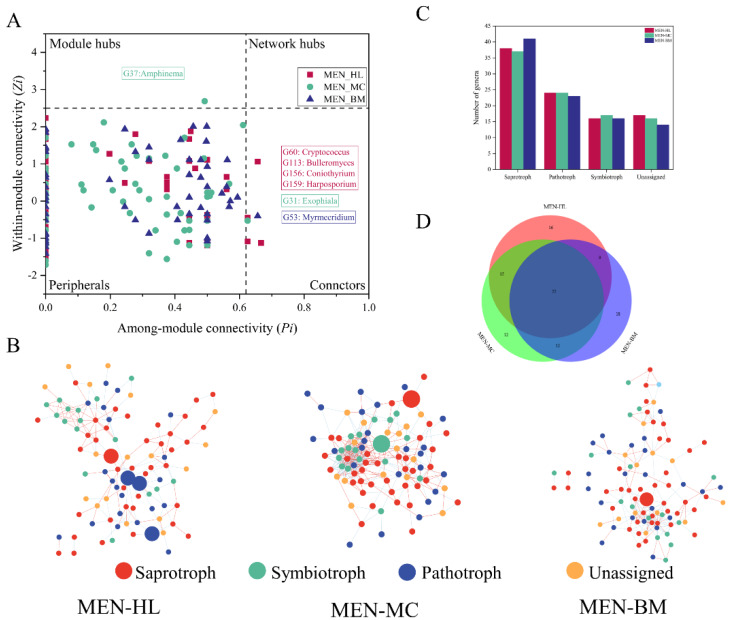
Network analysis of fungal communities in three wetlands. Panel (**A**) shows a Z–P plot of the topological roles of each fungal genus, the generalists (connectors and module hubs) in the network are labeled at the genus level. Panel (**B**) shows the fungal network in the three wetlands. Red lines show positive correlations, and blue lines show negative correlations. Node colors represent different trophic modes. The generalists are marked with a larger circle. Panel (**C**) shows the number of trophic modes in the three fungal ecological networks. Panel (**D**) shows a Venn diagram of fungi in the genus level among three fungal ecological networks. MEN-HL, MEN-MC and MEN-BM represent fungal phylogenetic molecular ecological network in the Huahu Lake wetland, the Mangcuo Lake wetland and the Baima Snow Mountain wetland, respectively.

Different nodes in the network play different topological roles [33]. The functional division of network nodes used the fast greedy algorithm. According to indicators of within-module connectivity (*Zi*) and between-module connectivity (*Pi*), nodes are divided into four categories: peripherals (*Zi* ≤ 2.5, *Pi* ≤ 0.62), connectors (*Zi* ≤ 2.5, *Pi* ≥ 0.62), module hubs (*Zi* ≤ 2.5, *Pi* ≥ 0.62) and network hubs (*Zi* ≥ 2.5, *Pi* ≥ 0.62) [31]. No network hubs appeared in the MEN of the three wetland fungal communities (Figure 4A,B). Four connectors appeared in the MEN of Huahu Lake wetland, which were *Cryptococcus, Bulleromyces, Coniothyrium* and *Harposporium*. According to trophic strategies annotation by FUNGuild, *Cryptococcus* and *Bulleromyces* are fungal parasites and *Harposporium* is an animal pathogen. The *Coniothyrium* guild is an undefined saprotroph. The Mangcuo Lake wetland had a module hub (*Amphinema,* an ectomycorrhizal saprotroph) and a connector (*Exophiala,* an undefined saprotroph). The Baima Snow Mountain wetland had only one connector belonging to the *Myrmecridium* genus, which was assigned as an undefined saprotroph.

Statistics on the nodes of the three networks are shown in Figure 4B. The nodes in the three wetland ecological networks are different, but 55 common nodes (genera) appeared in all three wetland fungal networks (Figure 4C). The fungal function annotation results (Figure 4D) showed that the trophic mode of the nodes in the fungal networks was very similar.

### 3.6. Geographic Sampling Location, Soil Properties and Fungal Distribution

Geographic sampling location and soil properties are important factors driving changes in fungal communities. The Mantel test was used to determine the environmental factors driving community change (Table S6). It showed that the environmental factors measured in this study had an extremely significant effect on the fungal community (*p* < 0.01), except for TP (*p* <0.05). After considering the correlation between environmental factors, the partial Mantel test showed that pH, AN and NN were the key environmental factors affecting fungal communities in wetlands (*p* < 0.01). Altitude was also an important geographic factor affecting fungal communities.

**Figure 5:**
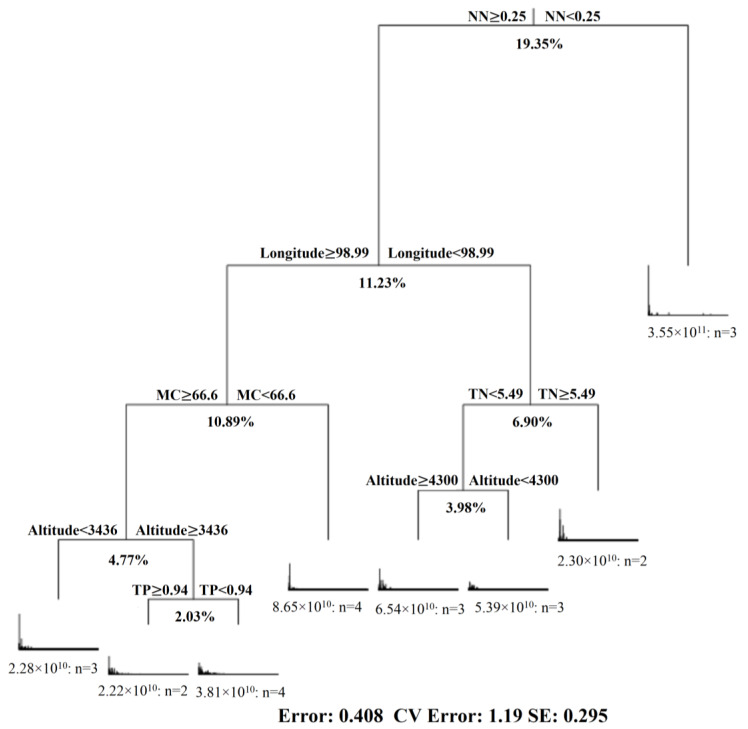
Multivariate regression tree (MRT) of fungal diversity data associated with geographic data (longitude, latitude and altitude) and soil physicochemical properties (pH, MC, TC, TN, TP, AN, NN and C:N ratio).

Multivariate regression tree analysis was conducted for the data from 24 samples from the three wetlands, including geographic data (latitude, longitude and altitude), soil physicochemical properties (pH, MC, TC, TN, TP, AN and NN) and fungal community information. A visualization tree generated by MRT analysis shows eight splits based on the characteristics of the sampling location and soil properties. The tree explained 59.15% of the variance of the standardized diversity indices. All data were divided into two groups based on nitrate content (NN), which accounted for 19.35% of the original data set change. Longitude explained 11.23% of the data set change, water content (MC) explained 10.89%, altitude explained 8.75% (3.98% and 4.77%) of the dataset, total nitrogen (TN) explained 6.90% and total phosphorus (TP) explained 2.03% of the variation in the original data set (Figure 5).

## 4. Discussion

This study examined three different alpine lakeside wetlands in Qinghai–Tibet Plateau and its edge and analyzed the composition, functional diversity and network composition of fungal communities in wetlands. The factors that drive community change were further analyzed.

The species richness of the fungal communities in three wetlands was all high, while the diversity was different (Figure 1 and Figure 2). Additionally, the three wetlands have different fungal communities with special ecological function (Figure 3). Among the three lakeside wetlands fungal communities, the relative abundance of the saprotrophic fungi was the highest, which is consistent with the research results in some forests and grasslands [17,42]. It is worth noting that the relative abundance of saprophytic fungi in the Baima Snow Mountain Lake wetland reached 80.78%, which is much higher than in the Mangcuo Lake wetland or Huahu Lake wetland, while the Baima Snow Mountain wetland had the lowest carbon content (11.27 ± 2.16 g/kg dry soil). The possible cause of this is that the saprophytic fungi in the alpine wetland have more moderate activity; the carbon content in soil is enough to sustain the metabolic activity for saprophytic fungi. The more likely reason is that the saprophytic fungi in the Baima Snow Mountain wetland are active in metabolism so that they consume most of the carbon in the soil. Saprotroph fungi in the three wetlands are composed mainly of undefined saprotroph, dung saprotroph, soil saprotroph, wood saprotroph and leaf saprotroph, and they are primary, secondary and tertiary decomposers in natural ecosystems [42,43]. The relative abundance of these trophic fungi in the three wetlands is different, indicating that saprophytic fungi mediated carbon cycle processes are diverse in different environments of alpine wetlands. There is a very significant negative correlation between symbiotic and saprophytic fungi in the three alpine wetlands (Table S3). Symbiotic fungi are mainly composed of endophyte and ectomycorrhizal fungi. Mycorrhizal fungi and saprophytic fungi depend to a certain extent on the same nutrient source and share a niche, which creates the potential for competition and antagonistic interference of the two taxa of fungi [44,45]. The competition will eventually affect the ecological function of the fungi in the wetland [45]. Pathotroph is mainly composed of the fungi of plant pathogens, animal pathogens and fungal parasites. The habitat of the Huahu Lake wetland is more suitable than the other two wetlands for pathogenic fungi, which has a higher relative abundance in this wetland. Corresponding to metrics based on species richness and taxonomic identity analysis results, the fungal communities in these three alpine wetlands have significant differences in trophic types. This indicates that in these alpine wetlands, fungi play different roles in wetland ecosystem processes. Fungal communities in alpine wetlands have rich functional diversity.

The topological relationships, interaction patterns and phylogenetic relationships of individual node shown potential interactions within fungal communities of three wetlands [32]. The ratio of the number of network edges to the number of nodes and average connectivity represents the complexity of the network. Positive ecological interactions might reflect commonly preferred conditions or cooperative behaviors, whereas negative ecological interactions would represent negative interactions and might represent competition [32,41]. Therefore, the fungal communities have different survival strategies in alpine wetlands (Figure 4 and Table S5). Among them, the interaction between fungi in Huahu Lake wetland is weak, and the fungi are more inclined to cooperate with each other. The interaction between fungi in the Mangcuo Lake wetland is the strongest, and there are more competition relationships than cooperation relationships among fungi. The competition among fungi in Baima Snow Mountain wetland is more intense, and the cooperation among fungi is less.

Each taxon of fungi plays a different role in the community, mapping to ecological network means that nodes have different topological functions [31,32,46]. According to the ZP plot, most of the fungi genera in the three wetlands belong to peripheral nodes. These fungi genera have few links with other fungi and always interact with fungi in the module. Connectors are essential species for connecting different modules [32]. In the Huahu wetland fungi network, four nodes (*Cryptococcus*, *Bulleromyces*, *Coniothyrium* and *Harposporium*) belong to connectors. The connectors in the fungal communities of the Mangcuo Lake Wetland and Baima Snow Mountain wetland are *Exophiala* and *Myrmecridium*, respectively. In the Mangcuo Lake wetland fungal ecological network, *Amphinema* is the only module hub and is a highly connected node in its own module [41]. Connectors and module hubs are generalists in fungal networks, and generalists are considered to be key taxa that have the greatest effect on microbial community structure and potential functions [41,47]. Therefore, the taxa driving the fungal communities in the three wetlands are different from the perspective of fungal biology classification. The ecological network analysis of fungal communities combined with functional annotation provides additional information on the functional diversity of wetland fungal communities (Figure 4). Based on the number of nodes, the three trophic modes in the three wetland fungal networks were similar. However, there were some functional differences between key species (generalists) in the three networks. Two of the generalists in the Huahu Lake wetland were fungus parasites: one was an animal pathogen, and one was an undefined saprotroph. The generalists of the Mangcuo Lake wetland were ectomycorrhizal and an undefined saprotroph. The generalist of the Baima Snow Mountain wetland was an undefined saprotroph. These results suggest that saprophytic nutrition was a keystone function in the three alpine wetlands, especially the Baima Snow Mountain wetland. However, pathotrophic fungi also had profound effects on the fungal community function in the Huahu Lake wetland, and ectomycorrhizal, which is in close cooperation with the plants in the Mangcuo Lake wetland, was also an essential fungal group in the wetland.

Contemporary environmental conditions and historical contingency (represented by geographic sampling location) are considered to be the two main factors affecting fungal communities in ecosystems [34,35,48]. Recent studies have suggested that geographical location and environmental conditions have a scale-dependent effect on microorganisms [49,50]. The three wetlands studied in our work are within the local scale (<1000 km) as suggested by previous study [48], environmental factors might be the main factor determining the variety of fungal communities. The partial Mantel test and multivariate regression analysis results show that the geographic factor (longitude and altitude) also had significant effects on the diversity of fungal communities. This result is different from the study of fungi in other areas, which may be due to the unique geographical isolation and habitat distribution of plateau areas making fungi community in plateau wetlands more affected by historical contingencies. This further shows that alpine wetlands are important areas for maintaining biodiversity.

When analyzing the influence of environmental factors on the fungal community, it was found that nitrogen (nitrate, ammonium and total nitrogen) was the most important environmental factor affecting the fungal community in alpine lakeside wetlands. A large number of reports show that the diversity of nitrogen uptake patterns of plants in the Qinghai–Tibet Plateau is relatively high, thereby enriching plant diversity in the plateau [51,52]. Plants are an essential source of nutrients for soil fungal communities, and fungal communities are closely related to plant communities [53,54,55]. Therefore, nitrogen is the most critical environmental factor driving changes in the fungal communities of the three alpine lakeside wetlands. The pH also affected the fungal community to a certain extent. The adaptation range of fungi to soil pH is wide [56], which may be the reason why the fungal community still has high abundance in the acid soil (pH = 5.7) of the Mangcuo Lake wetland. In addition, moisture content (MC) indirectly affects fungal communities by affecting soil pH, availability of nutrients and soil aeration. The average soil moisture content of the studied wetland was 58.25–164.77%, and the difference in moisture content also affected the fungal community composition and diversity in alpine wetlands.

## 5. Conclusions

In this study, we focused on the fungal communities of three alpine lakeside wetlands on the Tibetan Plateau and its edge. We used high-throughput sequencing data and microbial bioinformatics analysis methods to investigate the species diversity, functional diversity of fungi in wetlands and the corresponding fungal ecological network. We combined geographical information and soil properties to reveal the factors that drive changes in fungal communities. Our study results showed that although the studied wetlands were all located in cold high-altitude areas, the fungal species in the three wetland soils were abundant and the fungal community was also diverse in function and network structure. Saprophytic fungi were the dominant species in the three wetland soil environments. In some fungal communities, pathotrophic fungi (in Huahu Lake wetland) and symbiotic fungi (in Mangcuo Lake wetland) were also key microorganisms affecting the structure and potential functions of fungal community. In this study of alpine lakeside wetlands, contemporary environmental conditions (soil properties) and historical contingencies (geographic sampling location) jointly determined the fungal community diversity at a small spatial scale (<1000 km). These results may expand our knowledge of fungal biodiversity in alpine lakeside wetlands, which has not been widely studied.

## Figures and Tables

**Table 1:** Soil properties of samples from Huahu Lake Wetland, Mangcuo Lake Wetland and Baima Snow Mountain Wetland.

	pH	MC (%)	TC (g/kg)	TN (g/kg)	TP (g/kg)	AN (mg/kg)	NN (mg/kg)	C/N Rate
HL1	7.00	136.37	170.83	13.89	0.80	0.76	1.33	12.30
HL2	6.97	200.47	172.35	13.61	0.90	1.17	1.10	12.66
HL3	7.02	162.36	112.62	8.65	0.87	0.86	0.69	13.02
HL4	7.02	159.86	117.17	8.90	0.90	1.27	0.66	13.17
HL5	7.11	163.56	128.61	12.06	0.98	1.22	0.95	10.66
HL6	7.00	162.31	150.29	12.10	0.87	0.71	0.61	12.42
HL7	6.99	150.57	164.77	12.12	0.90	1.19	1.42	13.59
HL8	7.21	182.62	130.36	8.83	0.55	0.85	0.66	14.76
MC1	5.82	88.74	55.05	5.19	1.30	0.61	1.07	10.61
MC2	6.11	54.62	43.10	3.84	0.95	0.54	1.52	11.22
MC3	5.53	47.06	38.45	3.39	1.02	0.40	2.62	11.34
MC4	6.09	41.58	36.95	3.28	0.94	0.44	1.35	11.27
MC5	5.41	79.61	59.53	5.53	0.96	0.52	2.59	10.76
MC6	5.42	98.63	33.26	5.75	1.20	0.51	1.83	5.78
MC7	5.37	71.94	51.35	4.96	0.87	0.47	1.50	10.35
MC8	5.75	86.20	58.05	5.44	0.73	0.63	1.49	10.67
BM1	6.43	46.31	19.53	2.12	1.04	3.12	0.23	9.21
BM2	6.48	41.90	16.38	1.99	1.11	4.94	0.33	8.23
BM3	6.57	63.22	27.43	2.94	0.94	6.39	0.29	9.33
BM4	5.76	80.70	37.05	3.67	1.05	5.02	0.17	10.10
BM5	6.16	69.98	26.23	2.85	1.17	6.32	0.22	9.20
BM6	6.67	42.05	15.73	1.94	0.69	4.87	0.19	8.11
BM7	6.00	65.41	30.53	3.41	1.08	5.21	0.19	8.95
BM8	6.60	56.40	21.08	2.62	1.29	3.68	0.24	8.05

HL1, MC1 and BM1 represent the first sampling points of Huahu Lake wetland, Mangcuo Lake wetland and Baima Snow Mountain wetland, respectively. And so on. MC: moisture content, TC: total carbon, TN: total nitrogen, TP: total phosphorus, AN: ammonia nitrogen, NN: nitrate nitrogen.

## Supplementary Materials

The following are available online at https://www.mdpi.com/2076-2607/8/5/632/s1. Figure S1: Venn diagram shows the unique and shared OTUs between Huahu Lake wetland (HL), Mangcuo Lake wetland (MC) and Baima Snow Mountain wetland (BM), Table S1: Relative abundance of fungi at genus level in Huahu Lake wetland (HL), Mangcuo Lake wetland (MC) and Baima Snow Mountain wetland (BM), Table S2: Permutational multivariate analysis of variance (PERMANOVA) of fungal communities in Huahu lake wetland (HL), Mangcuo lake wetland (MC) and Baima Snow Mountain wetland (BM), Table S3: Statistics of OTU number and sequence number of fungi successfully assigned to guilds, Table S4: Pearson correlation of three trophic modes of fungi based on sequence numbers, Table S5: Topological properties of the empirical and random MENs of fungal communities at three wetlands, Table S6: Mantel and Partial Mantel test of altitude and environment factors and Fungal community of three wetlands.
