# Supplementary material for: Revealing Fungal Communities in Alpine Wetlands through Species Diversity, Functional Diversity and Ecological Network Diversity
Source: Microorganisms. 2020 Apr 27;8(5):632. doi: 10.3390/microorganisms8050632 (PMC7284966; doi:10.3390/microorganisms8050632)
Supplement: Supplementary file 1 [file microorganisms-08-00632-s001.pdf]

microorganisms

MDPI

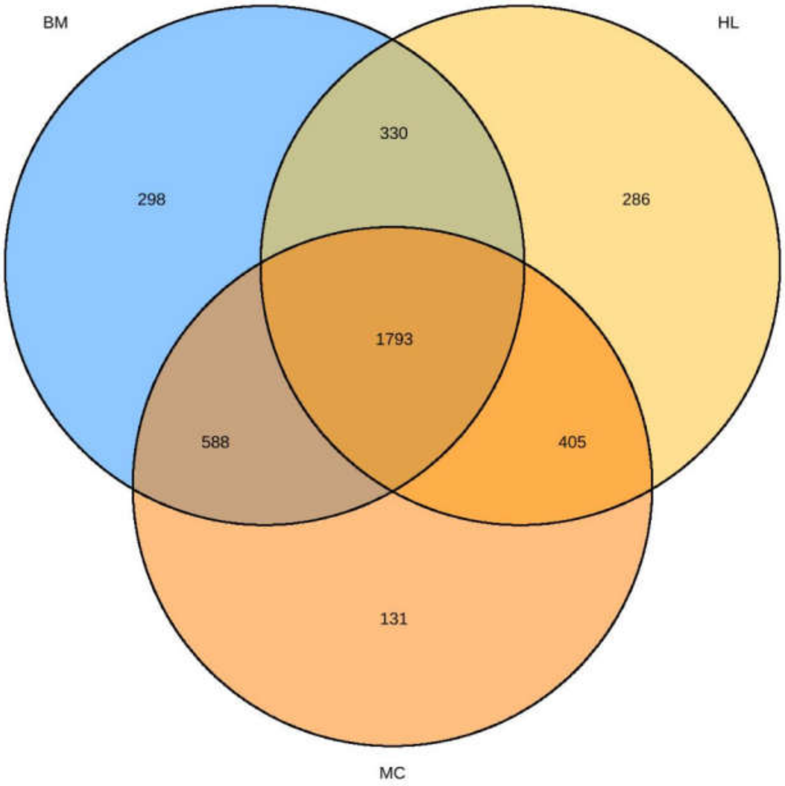

Figure S1. Venn diagram shows the unique and shared OTUs between Huahu Lake wetland (HL), Mangcuo Lake wetland (MC) and Baima Snow Mountain wetland (BM).

Table S1. Relative abundance of fungi at genus level in Huahu Lake wetland (HL), Mangcuo Lake wetland (MC) and Baima Snow Mountain wetland (BM).

Genus	HL	MC	BM
Unassigned	0.8555	0.7842	0.8487
others	0.0479	0.0410	0.0419
Mortierella	0.0051	0.0398	0.0265
Dimorphospora	0.0018	0.0424	0.0253
Solicoccozyma	0.0004	0.0013	0.0192
Neobulgaria	0.0002	0.0053	0.0117
Tomentella	0.0006	0.0009	0.0101
Inocybe	0.0005	0.0010	0.0072
Piloderma	0.0013	0.0231	0.0029
Sebacina	0.0067	0.0079	0.0015
Tricholoma	0.0121	0.0138	0.0015
Phialophora	0.0603	0.0023	0.0014
Russula	0.0044	0.0063	0.0011
Lactarius	0.0027	0.0049	0.0004
Coprinopsis	0.0003	0.0121	0.0003
Chaetosphaeria	0.0002	0.0134	0.0002

Table S2. Permutational multivariate analysis of variance (PERMANOVA) of fungal communities in Huahu lake wetland (HL), Mangcuo lake wetland (MC) and Baima Snow Mountain wetland (BM).

PERMANOVA	HL	MC	BM
HL		0.001	0.001
MC	7.64		0.001
BM	6.27	4.04	

The values of upper triangular matrices are the significance value (p-value). The values of lower triangular matrices are F-value.

Table S3. Statistics of OTU number and sequence number of fungi successfully assigned to guilds

		HL	MC	BM
OTU	Guild (OTU)	952	1093	1091
	Total (OTU)	2402	2538	2561
	G:T ratio (%)	39.63	43.07	42.60
Sequence	Guild (sequence)	478673	578897	562761
	Total (sequence)	763022	939462	877991
	G:T ratio (%)	62.73	61.62	64.10

Guild (OTU)/ Guild (sequence): OTU/sequence annotated by FUNGild software as a specific trophic type; Total (OTU)/ Total (sequence): Sum of OTUs/sequence that excluded unassigned OTUs/sequence and OTUs/sequence identified only at the kingdom level; G/T ratio: Number of successfully annotated OTUs/sequence as a percentage of total OTUs/sequence.

Table S4. Pearson correlation of three trophic modes of fungi based on sequence numbers

Pearson correlation	Saprotroph	Pathotroph
Pathotroph	−0.238	
Symbiotroph	**−0.909****	−0.188

significance of correlation coefficient (*$p \leq 0.05$, **$p \leq 0.01$).

Table S5. Topological properties of the empirical and random MENs of fungal communities at three wetlands

MEN	MEN-HL	MEN-MC	MEN-BM
Similarity threshold	0.81	0.78	0.8
Network size	95	94	94
Total links	167	365	206
Average connectivity	3.52	7.77	4.38
Average path distance	4.82	3.21	4.23
Average clustering coefficient	0.18	0.32	0.22
Modularity (no. of modules)	0.64(8)	0.4(5)	0.51(10)
Average path distance ± SD	3.615 ± 0.079	2.51 ± 0.037	3.11 ± 0.072
Average clustering coefficient ± SD	0.038 ± 0.013	0.20 ± 0.022	0.08 ± 0.016
Modularity ± SD	0.497 ± 0.014	0.254 ± 0.008	0.49 ± 0.014
Edge type (np)	81	199	131
Edge type (pp)	86	166	75

MEN-HL: Fungal phylogenetic molecular ecological network in Huahu lake wetland; MEN-MC: Fungal phylogenetic molecular ecological network in Mangcuo lake wetland; MEN-BM: Fungal phylogenetic molecular ecological network in Baima Snow Mountain wetland.

Table S6. Mantel and Partial Mantel test of altitude and environment factors and Fungal community of three wetlands

**Factors**	**Mantel Test**		**Partial Mantel Test**	
	r	***p***	***r***	***p***
pH	**0.567**	0.001	**0.341**	0.003
MC	**0.365**	0.001	−0.189	0.998
TC	**0.337**	0.001	−0.358	1.000
TN	**0.315**	0.001	−0.272	1.000
TP	**0.164**	0.018	0.113	0.064
AN	**0.353**	0.001	**0.314**	0.001
NN	**0.381**	0.001	**0.366**	0.001
C:N ratio	**0.305**	0.001	−0.043	0.695
Altitude	**0.632**	0.001	**0.3435**	0.001

MC: moisture content, TC: total carbon, TN: total nitrogen, TP: total phosphorus, AN: ammonia nitrogen, NN: nitrate nitrogen.
